# Telomerase inhibitors - TMPyP4, BIBR 1532 and imetelstat - alter the adhesion potential of breast cancer MCF7 and MDA-MB-231 cells that leads to impaired ability to form spheroids

**DOI:** 10.1080/19336918.2025.2571328

**Published:** 2025-10-13

**Authors:** Przemysław Kopczyński, Aleksandra Romaniuk-Drapała, Kinga Rygiel, Jacek Kujawski, Błażej Rubiś

**Affiliations:** a Centre for Orthodontic Mini-Implants, Department and Clinic of Maxillofacial Orthopedics and Orthodontics, Poznan University of Medical Sciences Poznan, Poland; b Department of Clinical Chemistry and Molecular Diagnostics, Poznan University of Medical Sciences Poznan, Poland; c Chair and Department of Organic Chemistry, Faculty of Pharmacy, Poznan University of Medical Sciences Poznań, Poland

**Keywords:** BIBR 1532, breast cancer, hTERT, imetelstat, telomerase, TMPyP4

## Abstract

We assessed the influence of telomerase inhibitors TMPyP4, BIBR 1532, or imetelstat on the ability of MCF7 and MDA-MB-231 breast cancer cells to form spheroids. TMPyP4 significantly impaired the adhesion potential and ability of both cell lines to form spheroids. BIBR 1532 treatment did not show any effect in MCF7 while it showed some effect in MDA-MB-231 cells, although this effect was less extensive comparing to TMPyP4. Application of Imetelstat provoked a dispersion effect in both cell lines but more single, separated distant cells were observed. Molecular docking and molecular dynamic studies showed that both BIBR 1532 and TMPyP4 exhibited affinity toward the structure of a G-quadruplex of human telomeric RNA (TERRA2 G4s) and the catalytic subunit of telomerase, hTERT. We showed that the use of telomerase expression/activity inhibitors to reduce the adhesive capacity and metastatic potential of breast cancer cells may play a significant role in anticancer strategy.

## Introduction

Cancer constitutes a global problem and is one of the leading causes of human mortality in the world [1]. The most common cancer in women is breast cancer. The increased risk of this cancer is mainly related to factors that are collectively referred to genetic and civilizational [2]. In spite of intensive prevention programs the disease rates are still raising mainly due to increased exposition to environmental risk factors, life style, stress and aging [3]. The main problem in achieving success in the field of effective oncological therapy is the high resistance of cancer cells to the chemotherapy drugs and, consequently, their resistance to pro-apoptotic signals [4]. Many studies to date (including ours) indicate that telomerase may be one of the key factors modulating both processes [5]. As it was shown, telomerase (mainly its catalytic subunit hTERT – human telomerase reverse transcriptase) is involved in migration and adhesion, which are the driving mechanisms of the metastasis [6]. During this mechanism, cell adhesion is decreased to separate cells from the parent tumor tissue and then increased to attach these cells to distant tissues [7]. Therefore, attempts are being made to identify targets for precision therapy (potentially hTERT is one of them), that would result in attenuated development of the disease or even its elimination via impairing cancer cell adhesion.

Before anticancer drugs are implemented, they are tested *in vitro* and *in vivo* (including animal experiments) to check the preliminary efficacy, toxicity and pharmacokinetics, and safety [8]. In monolayer cultures, cells have easy access to nutrients and oxygen, resulting in a uniform (to some extent) population of cells in terms of genotype and phenotype. It should be emphasized that cancer cells cultured in such conditions lack the complexity of the structure of a tumor growing *in vivo*, including vascularization and the presence of inflammatory cells [9]. Therefore, it is necessary to search for alternative experimental models that will facilitate assessment of the response of cancer cells to administered anticancer substances in conditions that resemble *in vivo* environment [10]. It seems that the approach based on 3D models could meet these expectations. It is recently gaining more attention since this approach more adequately recapitulates the *in vivo* conditions – at least when compared to classic 2D research models. Aggregates of cancer cells that are cultured *in vitro* show the characteristics of a tumor growing *in vivo* in the early phase of its growth and are therefore considered to be an intermediate form between cells from a single-layer culture and a tumor growing spontaneously [11]. Cell cultures in 3D, especially spheroids, in terms of morphology, consist of cells with a diverse phenotype, in the resting phase of the cell cycle, proliferating cells and cells with necrotic changes located in the center of the spheroid [12]. 3D cultures show a morphological similarity to tumors collected intraoperatively. This similarity is also reflected in the cell-cell interactions, intracellular signaling pathways and gene expression [13]. Consequently, the response of spheroids and tumors growing *in vivo* to the administered anticancer substances is comparable. Noteworthy, the IC50 (inhibitory concentration) values in a single layer in classic 2D cultures, is usually lower when compared to 3D systems [10]. Thus we decided to perform a study of the contribution of telomerase/hTERT to breast cancer cells metabolism in a spheroid 3D model of breast cancer in the context of functional assessment of cell-cell interactions.

Although cancer cells are derived from normal cells they show some distinct differences. Telomerase, an enzyme with reverse transcriptase activity that maintains the ends of chromosomes (telomeres), is induced and activated in ca 90% of cancer cases [14]. However, it does not occur in most normal cells [15]. Telomerase is therefore an attractive target for both diagnostics and therapy due to its expression profile [16]. As previously reported hTERT expression levels are observed in MCF7 and MDA-MB-231 cells – Luminal A and Normal-like/Tripple negative models, respectively [17]; the most common and most difficult to treat [18]. Due to its activity, telomerase restores telomeric ends leading to unlimited proliferative potential of breast cancer cells [19]. In vertebrates, telomeres are built of tandem repeats of the TTAGGG sequence, which are bound by a specialized group of protective proteins, collectively called the shelterin complex [20]. Further telomere protection structures are T-loops and G-quadruplexes, two tertiary DNA structures that protect the ends of telomeres and regulate their length [21]. Identification of the therapeutic agents targeting these regulatory factors may be a good approach to fighting cancer because telomerase expression/activity and telomere length are significantly correlated with cancer initiation and progression [22]. A significant correlation was found between enzyme activity and tumor size, lymph node status, and stage, and with tumor progression [23].

However, telomerase is also postulated to play some telomere-independent functions that are called noncanonical functions. As a result of numerous studies, it has been proven that cells expressing hTERT show increased adhesion to the extracellular matrix [6,24]. The adhesion process is particularly important in tumor progression and metastasis formation. During metastasis formation, cell adhesion is downregulated to separate cells from the primary tumor and then induced to nest the same cells to distant tissues [6]. Therefore, the aim of the study was to evaluate the effect of telomerase inhibitors TMPyP4, BIBR 1532 or imetelstat on the ability of MCF7 and MDA-MB-231 breast cancer cells to form spheroids in 3D culture.

The first compound, TMPyP4, is a potent inhibitor of human telomerase that binds strongly to DNA quadruplexes by stacking on the G-tetrads at the core of the quadruplex, resulting in telomerase inhibition [25–28]. However, it is postulated that TMPyP4 might not be limited to the influence on telomerase by G-quadruplex stabilization. As demonstrated, photodynamic therapy with TMPyP4 led to the formation of reactive oxygen species and changes in expression of genes involved in oxidative stress response in human hepatoma cell line HepG2 and human glioma cell line U251 [29]. Moreover, some reports suggest the complexity of TMPyP4 action in cancer cells including not only G4 stabilization but also a contribution to the regulation of expression of some genes engaged in cell metabolism, proliferation, and survival [30,31]

BIBR 1532 is a noncompetitive inhibitor of telomerase activity [32] that reveals antiproliferative effect on numerous leukemia cells while no effect on the proliferative capacity of normal hematopoietic progenitor cell was observed [33]. BIBR 1532 was shown to reduce colony-forming ability, induce telomere length shortening and cause chemotherapeutic sensitization via inhibiting telomerase activity in MCF-7/WT and melphalan-resistant MCF-7/MlnR cell lines [34]. BIBR 1532 demonstrated cytotoxic properties in a dose-dependent manner in T-cell prolymphocytic leukemia (T-PLL) [35], and in combination with carboplatin (a chemotherapeutic agent) eliminated ovarian cancer spheroid-forming cells in ES2, SKOV3, and TOV112D cell lines [36]. As reported, BIBR 1532 experiences ineligible pharmacokinetics [37]. Thus, numerous modifications of the compound to obtain new analogues were synthesized. Due to their inhibitory potential they showed strong antitumor effects against MCF-7 and A549 cancer cell lines including alteration of the cell cycle and apoptosis. Also, molecular docking studies were involved to evaluate their potentials upon variable structural modifications [37].

One of the most efficient solutions was offered by Geron that implemented Imetelstat (GRN163L, distributed and provided by Johnson & Johnson) that is a potent and specific telomerase inhibitor. That is so far the only drug of its class in clinical trials. On 6 June 2024, the Food and Drug Administration approved Imetelstat (Rytelo, Geron Corporation), an oligonucleotide telomerase inhibitor, for adults with low- to intermediate-1 risk myelodysplastic syndromes (MDS) with transfusion-dependent anemia requiring four or more red blood cell units over 8 weeks who have not responded to or have lost response to or are ineligible for erythropoiesis-stimulating agents (ESAs) [38]. Imetelstat binds to the telomerase RNA component with high affinity, inhibiting telomerase activity directly, rather than through antisense inhibition of protein translation [39]. Imetelstat has shown promising results in treating patients with hematological malignancies and solid tumors. Some studies have even reported synergistic effects when Imetelstat was used in combination with other anti-cancer drugs or radiation therapy [40]. Although, when applied in solid tumors, it usually requires a longer treatment time [41].

Importantly, due to critical role of telomerase in cancer, other studies aiming to identify new potential inhibitors of the enzyme were performed including proapoptotic compounds e.g., triazole derivatives [42], G4 stabilizers [43–47] small molecules capable of interfering with the assembly of telomerase complex [48] or hTERT expression downregulators [49]. All these approaches can be more significant if computational study is involved that was also demonstrated [50] showing how molecular docking and mtQSAR activity prediction experiments could identify new efficient structures exhibiting the most pronounced inhibitory effect on tumor cells. These methods enable assessment of the potential of specific and stable interactions of potential telomerase inhibitors.

## Materials and methods

### Cell culture

Two cell lines representing different molecular subtypes of breast cancer were enrolled in the study, i.e., (i) MCF7, luminal A (ER/PR+, HER2low, TP53WT), and (ii) MDA MB-231, basal-like subtype, also called triple-negative breast cancer (TNBC; ER/PR-, HER2-, TP53mut). MCF7 cell line, in comparison to the MDA-MB-231 cell line, is a poorly aggressive and non-invasive cell line. Overall, it is being considered to show low metastatic potential. The MCF7 (HTB-22) and MDA-MB-231 (HTB-26) cells were maintained in RPMI-1640 medium (Biowest, Nuaillé, France), supplemented with 10% fetal bovine serum (FBS) (Biowest, Nuaillé, France) at 37°C in an atmosphere of 5% CO_2_ and saturated humidity. Both cell lines were obtained from the American Type Culture Collection (ATCC; batch numbers: 17J001/30029090 and 11D011/30029090, respectively). As previously demonstrated, both cell lines express catalytic telomerase subunit while MCF7 shows higher hTERT protein accumulation than MDA-MB-231 [17]. Cells were routinely checked for *Mycoplasma* detection using DAPI staining.

### Cell treatment

Cell were treated for 72 hours with the tested telomerase inhibitors. For each cell line, Petri dishes (6 cm) containing 130,000 cells each were prepared for both cell lines. Then, specific inhibitor compounds were added at specific target concentrations (concentrations were adjusted referring to data obtained in our previous work [17] (the effect was time- and dose-dependent, but cell viability was reduced by no more than 30% in any of the concentrations applied after 72 h) and other literature data [51] as subcytotoxic i.e.:

- TMPyP4 (5 µM, 10 µM or 20 µM)
- BIBR 1532 (1 µM, 5 µM or 10 µM)
- Imetelstat (1 µM,5 µM or 10 µM)
- Mismatch negative control for Imetestat (1 µM, 5 µM or 10 µM).

After 72 h treatment, MCF7 and MDA-MB-231 cells were trypsinized from each plate and, after adding telomerase inhibitors at the target concentrations as specified above, transferred to 96-well Ultra-Low Attachment Multiple Well Plates (Corning ULA plates, Merck, Germany) at a density of 5x10^2^ cells/well in six repetitions for a given compound and concentration. The cells were observed and photos were taken every 24 h up to three days.

### Molecular docking and molecular dynamic studies

Initial structures of ligands were taken from the PubChem [52] database (PubChem CID: 9,927,531, 135,442,972, and 72,941,969 for BIBR 1532, TMPyP4, and Imetelstat, respectively). For each ligand the density functional theory (DFT) calculations were executed, and geometries were optimized using the Gaussian 16 C.01 program [53] at the B3LYP/6-31 G(d,p) level of theory [54]. For semiempirical calculations we used the Mopac 2016 program [55], PM7 functional and its implemented Mozyme module, and the previously described protocol [56]. The structure of a G-quadruplex of human telomeric RNA (TERRA2 G4s; the NMR PDB model 2kbp.pdb represents the ribonucleic sequence 5’-UAGGGUUAGGGU-3 ’) [57] or catalytic subunit of telomerase, TERT (3du6.pdb protein) [58], were selected as biological targets. The genetic algorithm (GA) method implemented in the AutoDock Vina program [59] was employed to provide the appropriate binding orientations and conformations of the compounds in pockets. Polar hydrogen atoms were added, and partial charges were assigned to the protein. A grid box of 40 Å size (coordinates: center _x = 0.278 or −10.414, centre_y = 0.14 or 16.05, centre_z = 0.195 or −29.702 for the 2kpb.pdb or 3du6.pdb, respectively) was defined to carry out the docking simulation. The outputs (*.pdbqt files) after docking procedure were visualized with the Chimera 1.13.1 package [60]. The interactions of analytes docked to the pocket were visualized with BIOVIA Discovery Studio Visualizer v24.1.0.23298 program [61]. For the molecular dynamics (MD) calculations, the GROMACS 2024.1 [62] was employed to simulate the solvated complexes. The Amber99SB-ILDN force field [63] was used to parameterize the protein and counter ions. The general GAFF force field [64] was utilized to represent the ligands and their topology with the help of Topolbuild 1.2.1 [62]. Finally, the complexes were inserted into the cubic water boxes using the TIP3P water model [65] (10 × 10 × 10 nm). The soluble complex consisted of one molecule of biological target, one ligand molecule, water molecules, and Na+ ions depending on the charge of the ligand. The soluble complexes were first minimized using the steepest descent scheme. Then, the minimized configurations were relaxed in NVT and NPT ensembles with 500-ps MD length per simulations. The complexes were restrained by NVT simulations using a small harmonic force. For the complexes free of restraints, we adopted NPT MD simulations. The relaxed system was then used as an initial conformation for 100-ns MD simulations. The time step used throughout the MD calculations was 1 fs.

### Data assessment

Results were expressed as representative pictures. All experiments were performed in six repeats.

## Results

### Effect of telomerase inhibitors on the cell-to-cell interactions and spheroid formation abilities

#### Effect of TMPyP4 on the cell-to-cell interactions and spheroid formation in MCF7 cells

**Figure 1.**
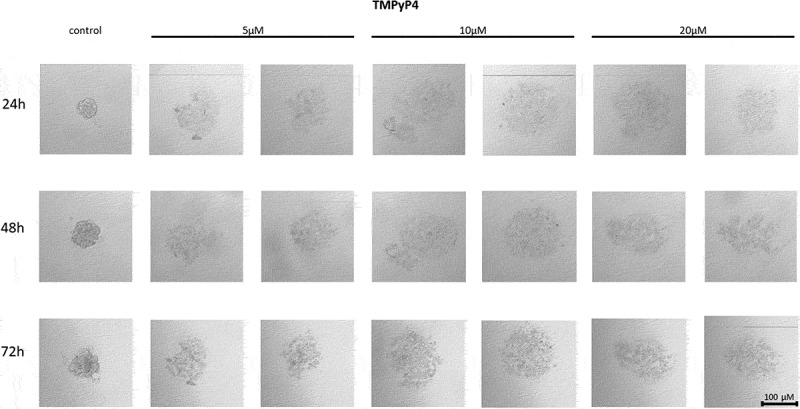
The effect of telomerase inhibitor, TMPyP4, on the ability of MCF7 to form spheroids. The experiment was performed in 6 replicates for every concentration and time interval. Incubation of the cells with the tested compound lasted 72 h, followed by 3D spheroid formation test for up to another 72 h, again in the presence of the inhibitor. Pictures were taken using CarlZeiss microscope, 10X. The scale bar shows 100 µm. Representative pictures are demonstrated.

The telomerase inhibitor TMPyP4 added at a concentration of 5 µM, caused a decrease in the ability of MCF7 to form a spheroid comparing to control, untreated cells (Figure 1). Importantly, as the concentration of the cationic porphyrin TMPyP4 increased, i.e. up to 10 µM or 20 µM, the cells were even more dispersed, without forming round-shaped spheroids. The cell dispersion effect was dependent on the concentration used, but did not increase with extended incubation time i.e. 24, 48 or 72 h.

#### Effect of TMPyP4 on the cell-to-cell interactions and spheroid formation in MDA-MB-231 cells

**Figure 2.**
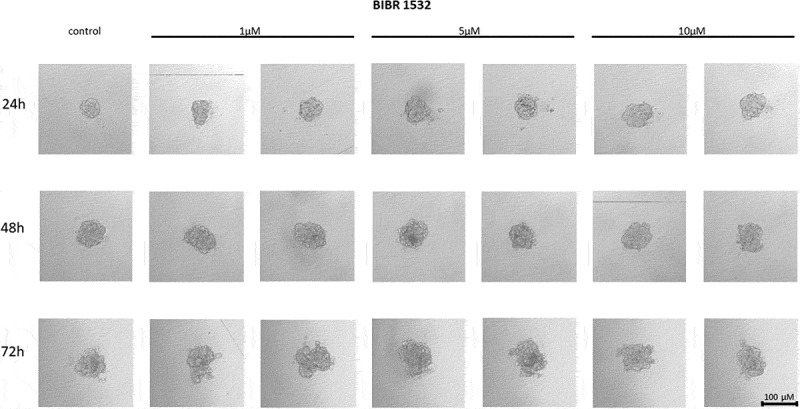
Effect of TMPyP4 inhibitor on the ability to form spheroids in MDA-MB-231 cells. The experiment was performed in 6 replicates for every concentration and time interval. Incubation of the cells with the tested compound lasted 72 h, followed by 3D spheroid formation test for up to another 72 h, again in the presence of the inhibitor. Pictures were taken using CarlZeiss microscope, 10X. The scale bar shows 100 µm. Representative pictures are demonstrated.

The TMPyP4 compound at each of the concentrations used decreased the cell-to-cell adhesion potential of MDA-MB-231 cells in a concentration-dependent manner. A time-dependent change in cell cluster formation was also observed. With each subsequent day, the state of cell dispersion increased, however, it may have been associated with the cell growth and proliferation which made the spheroid look bigger but, importantly, not that condensed as the control, untreated cells (Figure 2).

#### Effect of BIBR 1532 on the cell-to-cell interactions and spheroid formation in MCF7 cells

**Figure 3.**
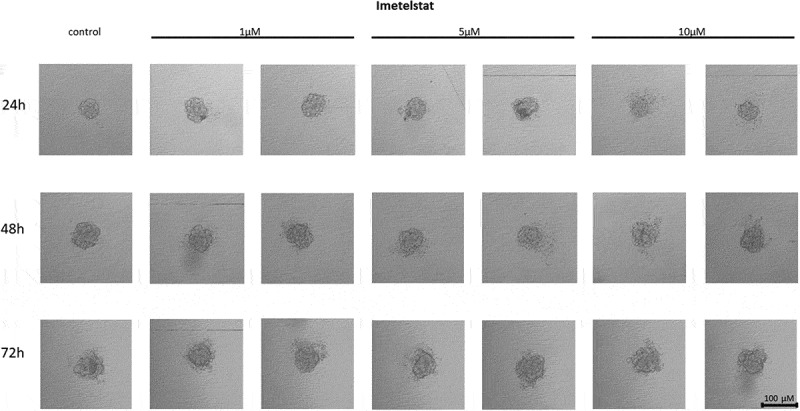
The effect of telomerase inhibitor, BIBR 1532, on the ability of MCF7 to form spheroids. The experiment was performed in 6 replicates for every concentration and time interval. Incubation of the cells with the tested compound lasted 72 h, followed by 3D spheroid formation test for up to another 72 h, again in the presence of the inhibitor. Pictures were taken using CarlZeiss microscope, 10X. The scale bar shows 100 µm. Representative pictures are demonstrated.

The non-nucleoside reverse transcriptase inhibitor BIBR 1532 treatment of MCF7 cells did not affect their ability to form spheroids at any of the concentrations used. No reduction in adhesion potential was observed in response to treatment with the above compound. No changes in spheroid formation were also observed with respect to the time of incubation of cells (Figure 3).

#### Effect of BIBR 1532 on the cell-to-cell interactions and spheroid formation in MDA-MB-231 cells

**Figure 4.**
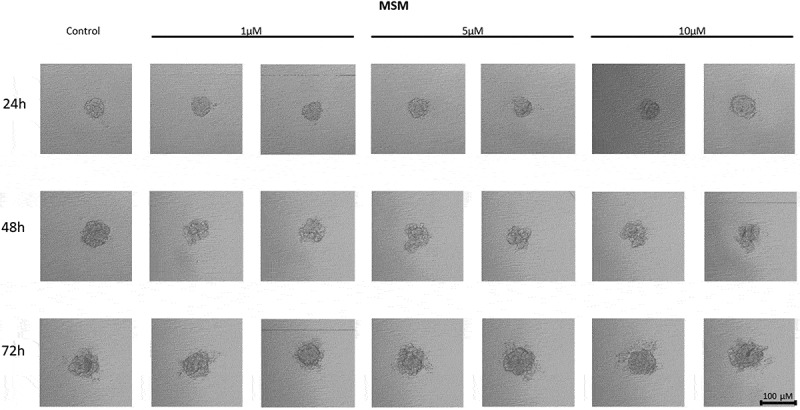
The effect of telomerase inhibitor, BIBR 1532, on the ability of MDA-MB-231 to form spheroids. The experiment was performed in 6 replicates for every concentration and time interval. Incubation of the cells with the tested compound lasted 72 h, followed by 3D spheroid formation test for up to another 72 h, again in the presence of the inhibitor. Pictures were taken using CarlZeiss microscope, 10X. The scale bar shows 100 µm. Representative pictures are demonstrated.

Treatment of MDA-MB-231 breast cancer cells with the telomerase inhibitor BIBR 1532 significantly influenced the ability of the cells to form three-dimensional clusters in culture. After applying the compound, the 3D structures did not form a compact architecture; on the contrary, after applying the compound, loose aggregates of cells creating an irregular shape were formed. With the time, the analyzed spheroids grew and increased in size, which is also visible in the control cells (Figure 4).

#### Effect of imetelstat on the cell-to-cell interactions and spheroid formation in MCF7 cells

**Figure 5.**
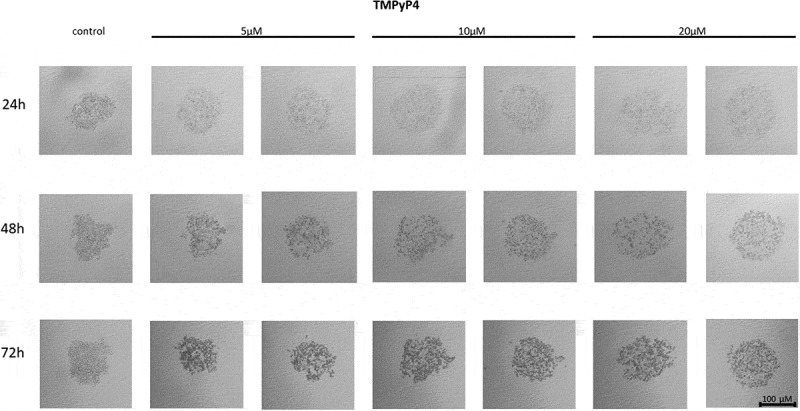
The effect of telomerase inhibitor, imetelstat, on the ability of MCF7 to form spheroids. The experiment was performed in 6 replicates for every concentration and time interval. Incubation of the cells with the tested compound lasted 72 h, followed by 3D spheroid formation test for up to another 72 h, again in the presence of the inhibitor. Pictures were taken using CarlZeiss microscope, 10X. The scale bar shows 100 µm. Representative pictures are demonstrated.

**Figure 6.**
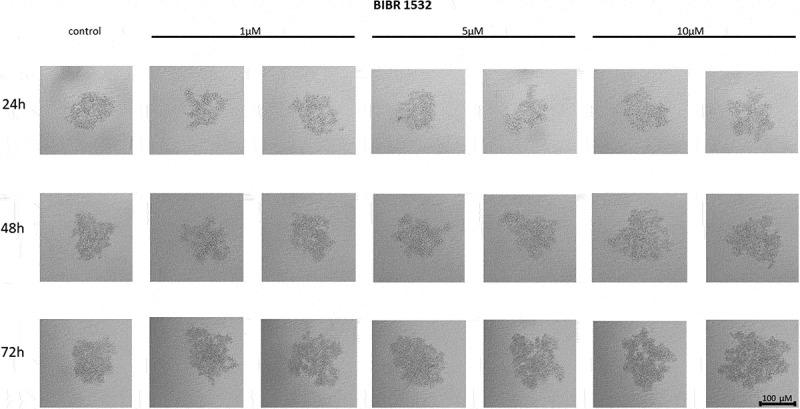
The effect of mismatch (MSM) negative control on the ability of MCF7 to form spheroids. The experiment was performed in 6 replicates for every concentration and time interval. Incubation of the cells with the tested compound lasted 72 h, followed by 3D spheroid formation test for up to another 72 h, again in the presence of the inhibitor. Pictures were taken using CarlZeiss microscope, 10X. The scale bar shows 100 µm. Representative pictures are demonstrated.

In MCF7 cells treated with the telomerase inhibitor imetelstat, a decrease in their adhesion capacity was observed in a concentration- and incubation-time-dependent manner. As shown in Figure 5, imetelstat was effective at a concentration of 5 µM and at the highest concentration used, namely 10 µM. In both of these concentrations, after 24 hours of incubation, cells were observed circulating around the formed spheroid. The compound used at a concentration of 1 µM did not significantly affect the ability of MCF7 cells to form spheroids. In turn, extending the incubation time resulted in greater cell dispersion. At the same time, no such effects were observed when cells were treated with a mismatch (MSM) control oligonucleotide (Figure 6).

### Effect of imetelstat on the cell-to-cell interactions and spheroid formation in MDA-MB-231 cells

**Figure 7.**
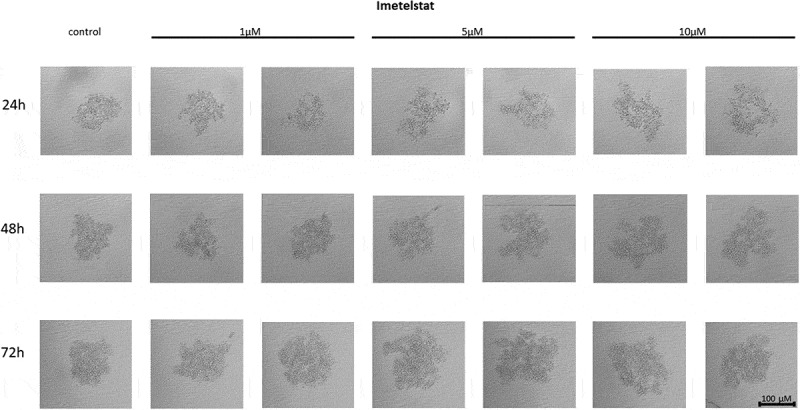
The effect of telomerase inhibitor, imetelstat, on the ability of MDA-MB-231 to form spheroids. The experiment was performed in 6 replicates for every concentration and time interval. Incubation of the cells with the tested compound lasted 72 h, followed by 3D spheroid formation test for up to another 72 h, again in the presence of the inhibitor. Pictures were taken using CarlZeiss microscope, 10X. The scale bar shows 100 µm. Representative pictures are demonstrated.

**Figure 8.**
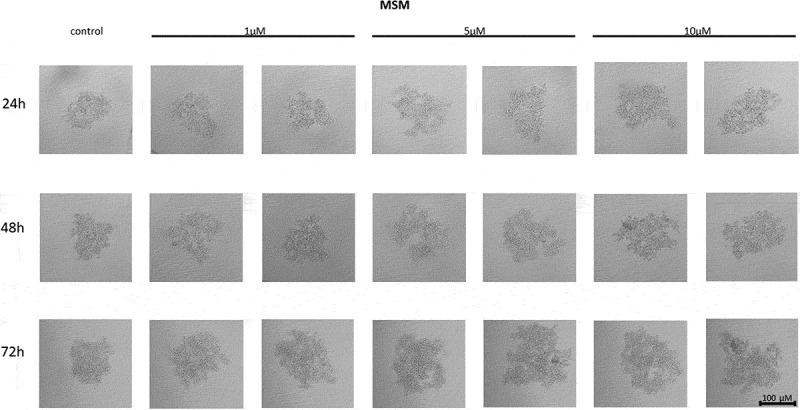
The effect of mismatch negative control on the ability of MDA-MB-231 to form spheroids. The experiment was performed in 6 replicates for every concentration and time interval. Incubation of the cells with the tested compound lasted 72 h, followed by 3D spheroid formation test for up to another 72 h, again in the presence of the inhibitor. Pictures were taken using CarlZeiss microscope, 10X. The scale bar shows 100 µm. Representative pictures are demonstrated.

Treatment of MDA-MB-231 breast cancer cells with imetelstat resulted in a decrease in their cell-to-cell adhesion and a decrease in their ability to form spheroids (Figure 7). The effect was directly proportional to the concentration – at a concentration of 10 µM, the highest degree of cell dispersion was observed compared to the control. The observed effect depended on the incubation time – the degree of dispersion increased with the incubation time. At the same time, no such effects were observed when cells were treated with a mismatch (MSM) control oligonucleotide (Figure 8).

All observed effects in the adhesion potential of studied breast cancer cells were compared and presented in Table 1.

**Table 1.** Contribution of telomere and telomerase modulators to the ability of MCF7 or MDA-MB-237 cells to form spheroids. All effects are referred to control, untreated cells except for imetelstat that is compared to mismatch oligonucleotide (negative control).

Compound	Cell line	Effect
TMPyP4	MCF7	Strongly dispersed spheroids
	MDA-MB-231	Strongly dispersed spheroids
BIBR 1543	MCF7	No visible effect
	MDA-MB-231	Slightly dispersed spheroid showing loosen tights
Imetelstat	MCF7	Major part of cells still forming a spheroid but some separate, very distant, single cells observed
	MDA-MB-231	Strongly dispersed spheroids
Mismatch/control	MCF7	No visible effect
	MDA-MB-231	No visible effect

## Molecular docking and molecular dynamic studies

**Figure 9.**
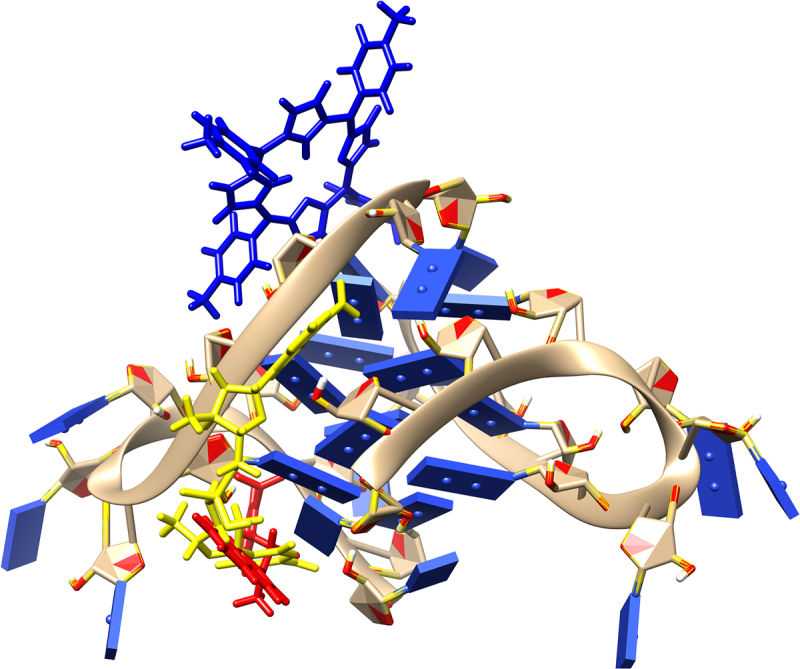
Docked ligands: BIBR 1532 (red), TMPyP4 (blue), Imetelstat_mod (yellow); first poses; protein 2kbp.pdb. For the docking protocol and visualization process we used the AutoDock Vina software and Chimera 1.13.1 package.

**Figure 10.**
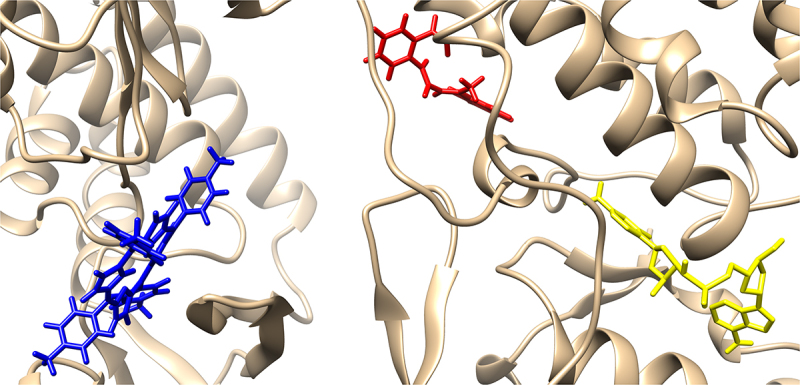
Docked ligands: BIBR 1532 (red), TMPyP4 (blue), Imetelstat_mod (yellow); first poses; protein 3du6.pdb. For the docking protocol and visualization process we used the AutoDock Vina software and Chimera 1.13.1 package.

**Figure 11.**
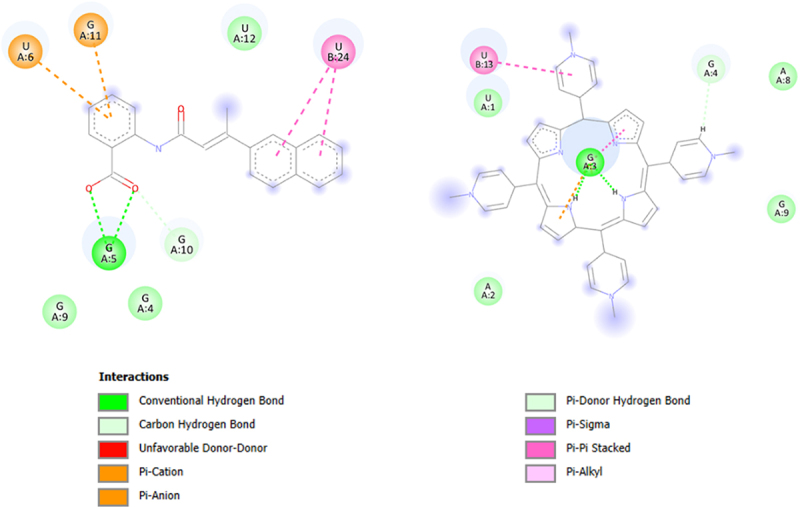
Type of interactions of BIBR 1532 (A) and TMPyP4 (B) within the 2kbp.pdb complex. The analysis was performed using the BIOVIA discovery studio visualizer v24.1.0.23298 program.

**Figure 12.**
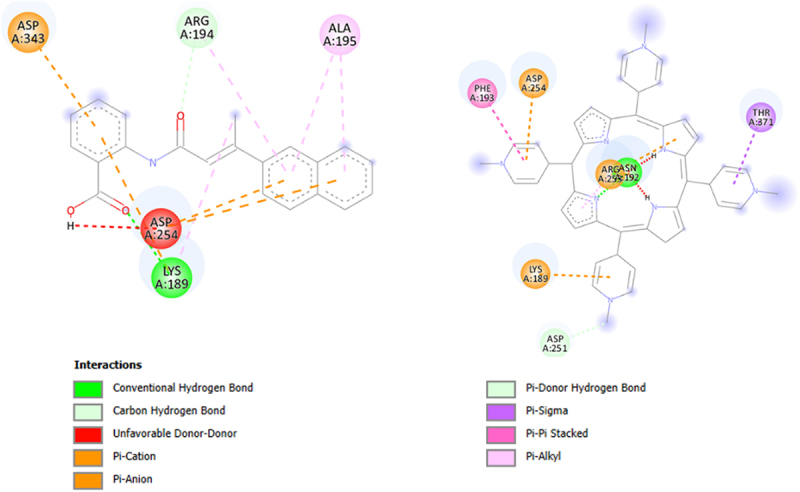
Type of interactions of BIBR 1532 (A) and TMPyP4 (B) within the 3du6.pdb complex. The analysis was performed using the BIOVIA discovery studio visualizer v24.1.0.23298 program.

Since BIBR 1532 and TMPyP4 were the subject of the *in vitro* test, with geometry previously optimized at B3LYP/6-31 G(d,p) level of theory in gaseous phase (Gaussian 16 C.01 program [53]) they were docked in the structure of a G-quadruplex of human telomeric RNA (TERRA2 G4s; the NMR PDB model 2kbp.pdb represents the ribonucleic sequence 5’-UAGGGUUAGGGU-3’) [57] or catalytic subunit of telomerase, TERT (3du6.pdb protein) [58]. In this case, The estimated binding affinity was as follows: −7.300, and −7.800 kcal mol^−1^ for BIBR 1532 and TMPyP4, respectively (2kbp.pdb; Figure 9) or −8.300, and −11.500 kcal mol^−1^ for BIBR 1532 and TMPyP4, respectively (3dud6.pdb; Figure 10). Due to large structure of the imetelstat, it was shortened (Imetelstat_mod) and undergo the docking protocol with following binding affinities: −8.600 (2kbp.pdb; Figure 9) or −12.800 kcal mol-1 (3dud6.pdb; Figure 10). It was also noticed that for both investigated compounds, π–π and π–cation interactions were observed (2kbp.pdb or 3dud6; Figure 11 or Figure 12, respectively). Such contacts were more dominant in the case of the TMPyP4 derivative. In contrast, for the BIBR 1532 derivative, hydrogen bonding interactions formed by the carboxyl moiety were particularly notable.

## Discussion

Cell-to-cell interactions can be significantly affected by numerous factors including major tumor environment (TME) component – extracellular matrix (ECM). Its major part is collagen that is crucial in tumor cell infiltration, expansion, and distant metastasis during cancer progression [66]. Since it is essential for preserving tissue architecture and supporting crucial cellular functions like proliferation and differentiation (tumor growth), cell invasion, and metastasis, it became an interesting target in cancer therapy strategy [67]. MCF7 and MDA-MB-231 cells are characterized as cells corresponding to two different breast cancer types (luminal A (ER/PR+, HER2low, TP53WT), and basal-like subtype, respectively). A microfluidic platform-based studies revealed a significant difference between MCF 7 (low) and MDA-MB-231 (high) invasive potential of these breast cancer cells [68] that reflected the receptor and ECM content. Noteworthy, all the components of ECM (including collagens, fibronectins, hyaluronic acids, laminins and proteoglycans) were shown to affect the efficiency of chemo-, radio-, and immunotherapy [69]. Some studies reveal that the content of specific type of collagen in the ECM may significantly contribute to aggressiveness of breast cancer cells through the induction of MMP-9 mRNA and this is one of the factors that justifies different characteristics of different breast cancer cells, including the difference between MCF7 and MDA-MB-231 cells [70]

Literature data indicate the involvement of hTERT in the process of adhesion and migration of cancer cells [6]. These processes, in turn, constitute an important element in the development and spread of cancer cells. The regulation of cancer cell adhesion and migration is complex and involves both interactions between proteins (e.g. cell-cell adhesion molecules, cadherins, integrins, MAPKs – mitogen-activated kinases, receptor tyrosine kinases, cytoskeletal proteins) and the modulation of various signaling pathways, which are known to control cell proliferation, survival and differentiation [71]. Telomerase catalytic subunit hTERT is perceived as one of the factors that can promote cell adhesion and migration by influencing the expression of cell adhesion-related genes, but the exact pathway mediating these mechanisms is still unknown [17]. As we previously demonstrated breast cancer treatment with TMPyP4 or BIBR 1532 showed some telomerase activity-independent effects also when no cytotoxic effect nor sensitization of MCF7 and MDA-MB-231 to doxorubicin was observed. Namely, these two compounds application led to modulation of B1 integrin, FAK (including p-FAK Y397 and p-FAK Y576/577) and paxillin (including p-paxillin) in breast cancer cells [17]. That in turn suggested that telomerase/hTERT targeting might substantially contribute to a significant modulation of cancer cell dissemination/metastasis. Interestingly, our studies showed significant differences in the structure of spheroids formed by cells of individual cell lines, with those formed by MCF7 cells being more compact than MDA-MB-231. This may be because MDA-MB-231 cells are characterized as more invasive than MCF7 suggesting different basal adhesion and migration properties.

Numerous studies aim to evaluate the effects of targeting cancer cells for migration and/or adhesion. They include, among others the cationic porphyrin TMPyP4 to inhibit telomerase in human breast cancer cells [72]. The putative action of TMPyP4 is not limited to its effect on G-quadruplex stabilization. It was shown that TMPyP4 photodynamic therapy led to the formation of reactive oxygen species and changes in the expression of genes involved in the response to oxidative stress [73]. Additionally, TMPyP4 affects the regulation of the expression of some genes involved in cancer cell metabolism, proliferation and survival [17]. These premises initiated studies aimed at revealing how compounds with such biological potential can be used in cancer therapy. Particularly important is the fact that they can cause biological effects in a telomere-independent manner [17]. Our experiment showed that the cationic porphyrin TMPyP4 affected the ability to form spheroids in both analyzed breast cancer cell lines from the lowest concentration used, i.e., 5 µM, and the effect was directly proportional to the concentration (10 and 20 µM, respectively). Breast cancer cells of both lines formed dispersed three-dimensional structures at the highest inhibitor concentration, suggesting a weakening of adhesive properties. The study showed that in both cell lines, the effect of cell dispersion was dependent on the concentration of the inhibitor used, and the ability to form spheroids in MDA-MB-231 cells was also dependent on the incubation time. It may result from the fact that the cationic porphyrin TMPyP4 stabilizes DNA guanine quadruplexes, inhibiting telomerase activity and decreasing gene expression (e.g., c-MYC) [30]. It also reduces the level of hTERT transcripts, suggesting two mechanisms of action. Some studies demonstrated that TMPyP4 could reduce the tumor growth rate in xenograft models and prolong the survival of laboratory animals [30]. As shown TMPyP4 (at a concentration of ≤0.5 µM) was capable of inhibiting hTERT expression and telomerase activity in cells of non-small cell lung cancer A549, cervical cancer HeLa, and osteosarcoma cells (U2-OS and SAOS-2) leading to increased cell migration and adhesion of the cells to the matrix. In contrast, higher concentrations (≥2 µM) slowed down cancer cell proliferation and increased induction of cell death [74]. In turn, treatment of MCF7 and MDA-MB-231 cells with the cationic porphyrin TMPyP4 significantly inhibited cancer cell migration and adhesion [17]. Similarly, research conducted by Liu et al. showed that the induced expression of hTERT increased cancer cells’ adhesion and migration potential. The experiment also showed that hTERT expression promoted cell adhesion and migration independently of telomerase activity [6]. That in turn correlates with our results showing decreased spheroid formation after TMPyP4 treatment in MCF7 and MDA-MB-231 cells.

Another telomerase targeting approach is based on using inhibitors that bind non-competitively to telomerase. One well-documented synthetic agent, BIBR 1532, belongs to the non-nucleoside reverse transcriptase inhibitors (NNRTIs) and can effectively bind to the enzyme and block its activity [32]. The specific mechanism for NNRTI inhibitors is not fully understood, although there are two main theories that have been generally accepted. The first states that BIBR 1532 binds to a binding site sufficiently close to the origin of replication to block the replication binding site, thereby inactivating telomerase [32]. Second, BIBR 1532 causes a conformational change in the telomerase structure upon binding with it, which then blocks the activity of the replication binding site [32]. In our study BIBR 1532 was used to assess the adhesion of MCF7 and MDA-MB-231 breast cancer cells at concentrations of 1, 5, and 10 µM, and it did not affect the cell-cell adhesion potential of MCF7 line cells. Despite incubation with the compound, the cells’ morphology did not change compared to control cells. However, studies carried out on MDA-MB-231 cells showed the formation of scattered, irregular cell aggregates and the effect of the telomerase inhibitor BIBR 1532 in an incubation time-dependent manner. Some studies revealed that treatment of MCF7 and MDA-MB-231 cells with a combination of arsenic trioxide (ATO) and BIBR 1532 inhibited the proliferation and colony-forming ability of breast cancer cells. The synergistic effect of ATO and BIBR 1532 resulted in decreased hTERT expression [74]. In combination dosing studies of the widely used breast cancer chemotherapeutic agent paclitaxel (limited success due to drug resistance) and BIBR 1532, it was shown that the drug combination inhibited MCF7 cell colony formation in a dose-dependent manner [75]. BIBR 1532 is supposed to act in a concentration-dependent manner and inhibit telomerase action by decreasing the expression of c-MYC and hTERT [76]. Interestingly, combination of BIBR 1532 and DOX did not show any significant effect in hTERT protein accumulation in MCF7 cells while in MDA-MB-231 cells the catalytic telomerase subunit was repressed after treatment with BIBR 1532 (48 and 72 h) as well as DOX (48 h) [17]. That observation was confirmed in our study showing decreased spheroid formation after BIBR 1532 treatment in MCF7 and MDA-MB-231 cells.

Another known and tested inhibitor that strongly inhibits telomerase is imetelstat (GRN163L) [77]. It was found to affect not only cancer cells, but presumably also cancer stem cells (CSCs) [78]. When breast cancer cell lines were treated with imetelstat *in vitro*, telomerase activity was inhibited in most tumor cells and CSCs. Additionally, imetelstat treatment reduced the fractions of CSCs present in breast cancer cell lines [78]. Differences between expression levels, telomerase activity or telomere length in these cell lines did not correlate with increased CSC sensitivity to imetelstat [78]. This suggests that the mechanism of action on the CSC subpopulation is independent of telomere shortening [78]. Imetelstat, was shown to bind the hTR RNA messenger component of telomerase, and consequently to stop telomere elongation. Studies conducted in 63 non-small cell lung cancer (NSCLC) cell lines showed that imetelstat inhibited the ability of cells to form colonies [79]. It was also shown to prevent telomere elongation and potent competitive inhibition of telomerase activity [80]. Interestingly, studies on the effect of imetelstat on CSC subpopulations indicate that the mechanism of action of imetelstat can be independent of telomere shortening [78]. We showed that imetelstat decreased in the adhesion capacity of MCF7 (including cell-to-cell interactions and spheroid formation) in a concentration- and incubation-time-dependent manner. Imetelstat was effective at a concentration of 5 µM and at the highest concentration used, i.e., 10 µM. Similarly, the studies performed on MDA-MB-231 cells demonstrated the inhibitory effect of imetelstat on cell-cell adhesion even at the lowest concentration used, i.e., 1 µM, and this effect was dependent on the incubation time.

Given that, for both ribonucleic sequence (2kbp.pdb) and hTERT protein (3du6.pdb), the modified form of Imetelstat exhibited a significantly higher absolute binding affinity, and considering that Imetelstat itself is already commercially available, further considerations – from the standpoint of computational chemistry – focused exclusively on BIBR 1532 and TMPyP4. As a result, it turned out that, for both biological targets, the binding affinity of the TMPyP4 derivative was more negative compared to BIBR 1532. In the next step, we focused on the assessment of enthalpy changes of the interactions of BIBR 1532 and TMPyP4 (ΔH_int_) in the 2kbp.pdb and 3dud6.pdb pocket. In this evaluation, we considered values of final heat of formation (HOF) under standard conditions using *Mopac 2016* program [55], PM7 functional and its implemented Mozyme module, using the previously described protocol [56]. The application of the thermodynamic cycle of Raha and Merz led to the values of the ΔH_int_ as follows: −75.060 and −1027.550 kcal mol^−1^ for BIBR 1532 and TMPyP4, respectively (2kbp.pdb) or −149.920 and −105.350 kcal mol^−1^ for BIBR 1532 and TMPyP4, respectively (3du6.pdb). In turn, with respect to the catalytic subunit of telomerase, TERT, TERT the resulted data provide evidence that BIBR 1532 best fitted in the 3du6.pdb pocket.

**Figure 13.**
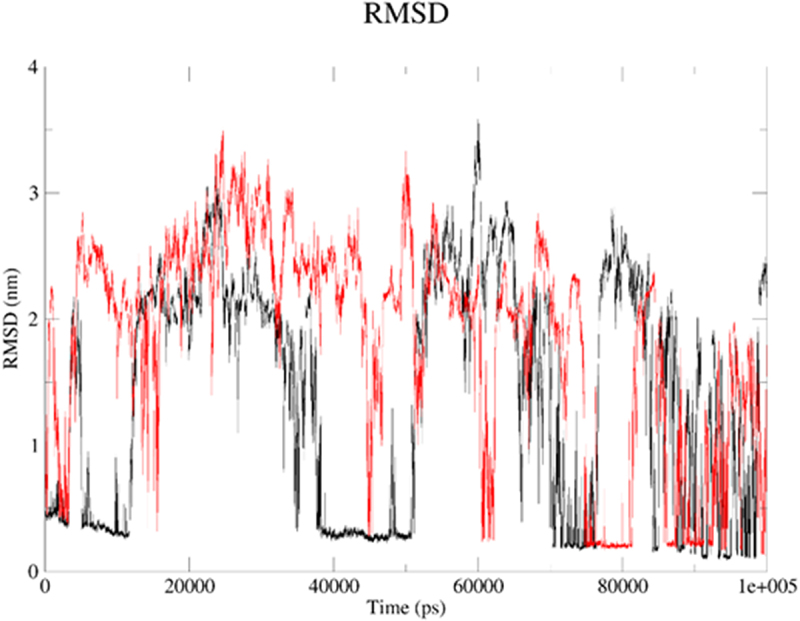
The RMSD plot for the backbone within ligand–2kbp.pdb complex: BIBR 1532 (black), and TMPyP4 (red). The MD calculations were caried out using the GROMACS 2024.1 software.

**Figure 14.**
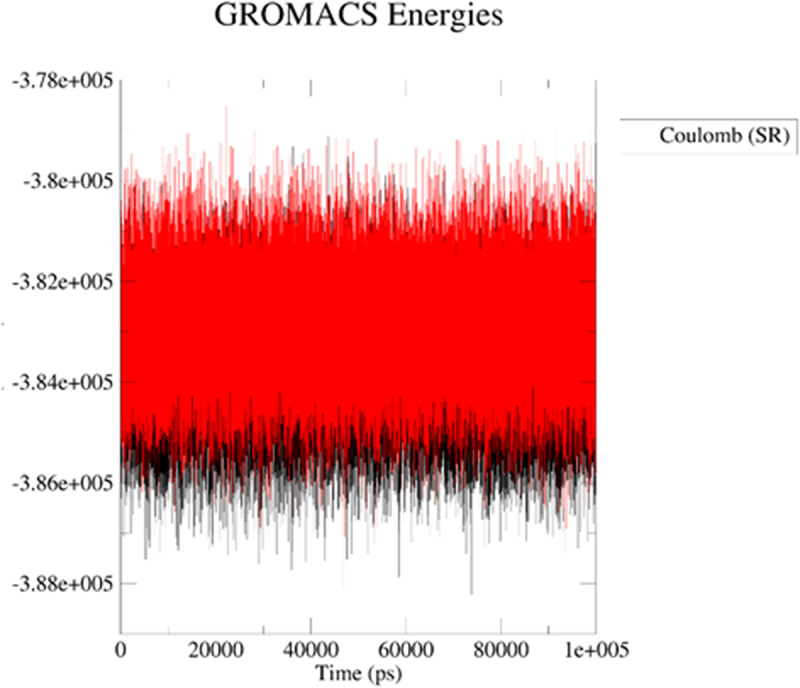
Energy plot for Coulomb (electrostatic) interactions for the ligand–2kbp.pdb complex during the productive phase: BIBR 1532 (black), and TMPyP4 (red). The MD calculations were caried out using the GROMACS 2024.1 software.

**Figure 15.**
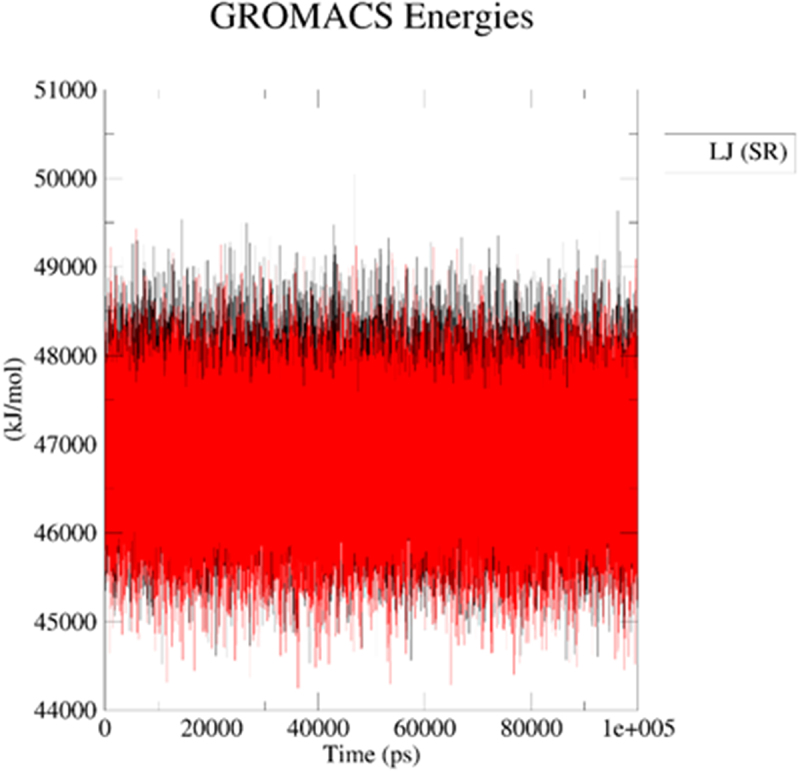
Lennard – Jones potential for the ligand–2kbp.pdb complex during the productive phase: BIBR 1532 (black), and TMPyP4 (red). The MD calculations were caried out using the GROMACS 2024.1 software.

**Figure 16.**
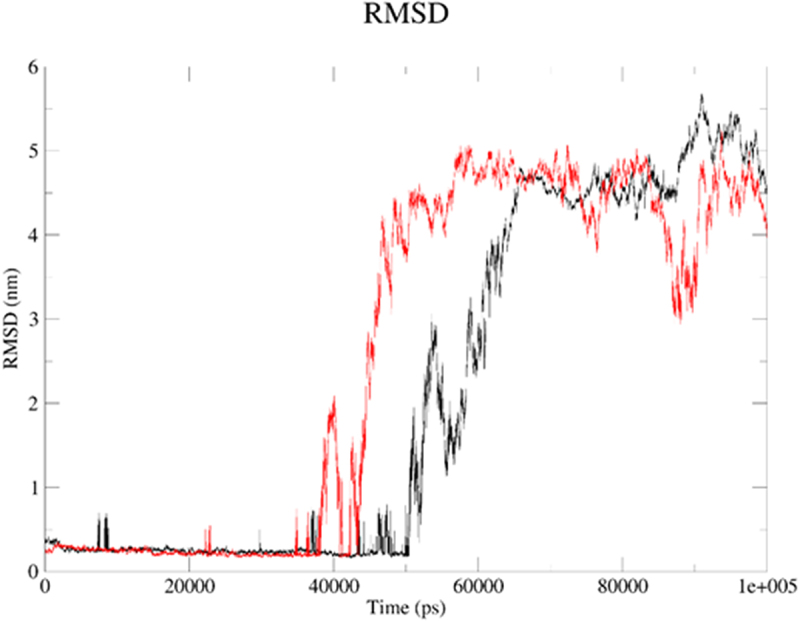
The RMSD plot for the backbone within ligand–3du6.pdb complex: BIBR 1532 (black), and TMPyP4 (red). The MD calculations were caried out using the GROMACS 2024.1 software.

Next, we applied molecular dynamics (MD) to explore stability of analytes in the 2kbp.pdb or 3du6.pdb pocket. For this purpose, the *GROMACS 2024.1* [62] was employed to simulate the solvated complexes (cubic water boxes). The RMSDs of backbone atoms (with the presence of ligands BIBR 1532 or TMPyP4) were calculated with respect to the initial configuration. The time evolution of RMSD values of the backbone in the complexes are shown in Figure 13 (2kbp.pdb) and Figure 16 (3du6.pdb). With respect to 2kbp.pdb, it was demonstrated that the RMSD parameter of the macromolecular cavity for both BIBR 1532 and TMPyP4 fluctuated between approximately ca 0.3 and 3.5 nm (ca 3 to 35 Å). Therefore, it can be concluded that the stability of the studied compounds in complex with 2kbp.pdb was quite similar. In this context, the stability of the complex involving BIBR 1532 was slightly more influenced by hydrogen bonding (Figure 14), whereas in the case of TMPyP4, van der Waals interactions (Lennard-Jones potential, Figure 15) played a more prominent role.

**Figure 17.**
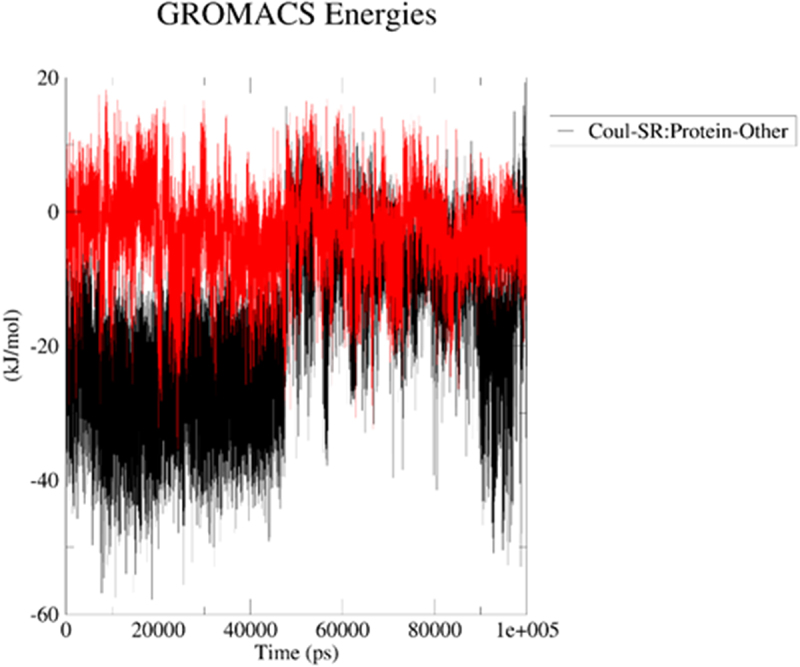
Energy plot for Coulomb (electrostatic) interactions for the ligand–3du6.pdb complex during the productive phase: BIBR 1532 (black), and TMPyP4 (red). The MD calculations were caried out using the GROMACS 2024.1 software.

**Figure 18.**
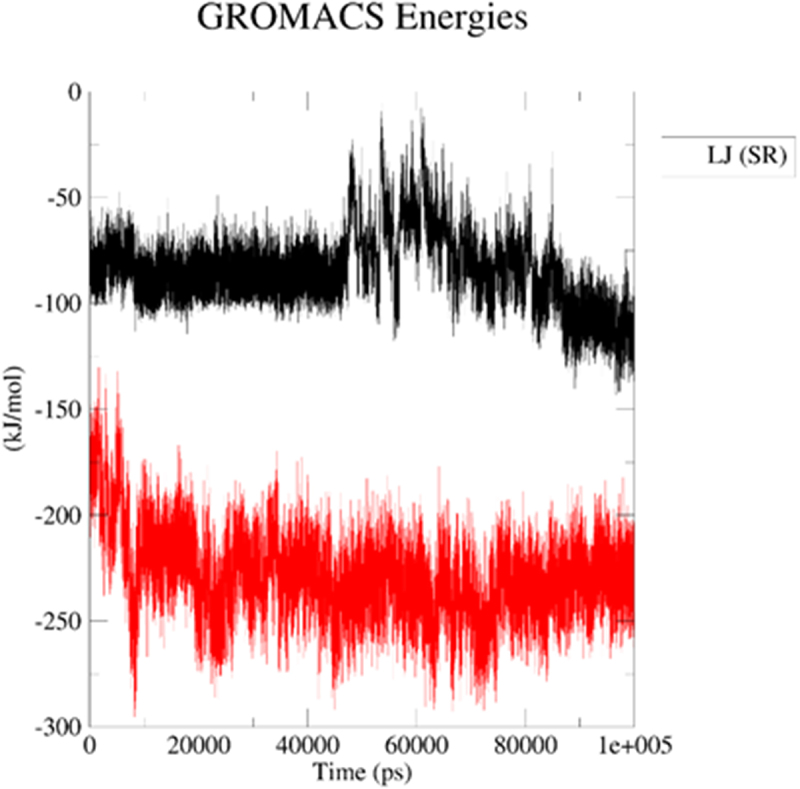
Lennard – Jones potential for the ligand–3du6.pdb complex during the productive phase: BIBR 1532 (black), and TMPyP4 (red). The MD calculations were caried out using the GROMACS 2024.1 software.

In case of 3du6.pdb protein, generally, ligands remained the backbone stable (regarding their influence on protein) for ca 50.3 (BIBR 1532) or 39.6 (TMPyP4) ns in their positions with RMSD oscillating at ca 3.00 Å (Figure 16); then, the RMSD curve moved upward and oscillated at 41.90–55.50 (BIBR 1532) or 29.80–50.60 (TMPyP4) Å, which clearly indicates that these systems were quite stable during the first 50.3 (BIBR 1532) or 39.6 (TMPyP4) ns. In the interactions of BIBR 1532 with 3du6.pdb, Coulombic interactions (hydrogen bond contacts) were significantly more favored (Figure 17). In contrast, the macrocyclic compound TMPyP4 with 3du6.pdb predominantly formed contacts governed by the Lennard-Jones potential (Figure 18).

## Conclusions

Our observations, together with other reports, demonstrate that telomerase inhibitors reduce the cell-cell adhesion potential of breast cancer cells, which may contribute to the modulation of their metastatic potential. Experiments showing the effect of telomerase/hTERT/telomeres targeting or application of telomerase inhibitors that act also in a telomere-independent manner, suggest their anticancer potential. Experiments performed in 3D culture demonstrate that the induced dispersion of cancer cells after the use of telomerase inhibitors may increase the area of action of the cytotoxic chemotherapy agent, that may affect the observed cytotoxicity effects of adjuvant therapy agents. However, since the results depend on the used agent and treated cell line, these studies require further analysis, taking into account the mechanisms associated with the ability of cancer cells to be released from the tumor into the bloodstream and the possibility of initiating new sites for cancer cell development. Noteworthy, the effect of the different telomerase inhibitors was not the same which may reflect their different mechanisms of action. Our data were demonstrated as a preprint [81].

In summary, it can be concluded that both BIBR 1532 and TMPyP4 exhibited affinity toward the structure of a G-quadruplex of human telomeric RNA (TERRA2 G4s) and the catalytic subunit of telomerase, TERT. However, the potential for interaction with the 3du6.pdb protein appeared to be more pronounced. In the studied complexes, BIBR 1532 demonstrated a stronger tendency to form hydrogen bonds, whereas TMPyP4 predominantly established contacts governed by the Lennard-Jones potential.

## Data Availability

All data supporting this study are openly available from Prof. Rubis.
